# Early experience of avacopan for ANCA-associated vasculitis in an Australian paediatric population: a case series

**DOI:** 10.1016/j.ero.2026.100205

**Published:** 2026-06-22

**Authors:** Anne Sage, Jonathan Akikusa, Sai Min Htay, Cassandra Cardenas-Brown, Gowri Manoharan, Anne Durkan, Nicholas Larkins

**Affiliations:** 1 Department of Rheumatology, Perth Children’s Hospital, WA, Australia; 2 Department of Rheumatology, Royal Children’s Hospital, VIC, Australia; 3 Department of Rheumatology, Sydney Children’s Hospital Network, NSW, Australia; 4 Department of Rheumatology, Queensland Children’s Hospital, QLD, Australia; 5 Department of Rheumatology, Royal Perth Hospital, WA, Australia; 6 Department of Nephrology, Sydney Children’s Health Network, NSW, Australia; 7 Department of Nephrology, Perth Children’s Hospital, WA, Australia

## Abstract

Avacopan, a complement inhibitor targeting C5a receptor, is a novel and effective agent in the treatment of antineutrophil cytoplasmic antibody (ANCA)-associated vasculitis (AAV) in adults. However, paediatric data of efficacy and safety are scarce. This report outlines our nationwide experience with avacopan for AAV in 8 adolescents. Avacopan was used as an adjunct following induction with glucocorticoids, rituximab, and/or cyclophosphamide at a median of 2 months postdiagnosis. There was good clinical improvement in 5 of the patients with an ability to wean steroid therapy; however, for 3, the disease progressed to end-organ damage despite avacopan introduction. There were no significant adverse events or serious infections reported with follow-up of a median of 6 months of avacopan use. The efficacy for most and short-term safety in all our cohort of avacopan is reassuring in the infancy of paediatric use and supports the need for further research in this orphan disease.

## INTRODUCTION

Antineutrophil cytoplasmic antibody (ANCA)-associated vasculitis (AAV) are a group of small- to medium-sized vessel vasculitides that often are multisystemic. Childhood-onset AAV (c-AAV) specifically, is an ultrarare and orphan disease with a female preponderance, unlike in adults [1]. Granulomatosis with polyangiitis (GPA) is the most common paediatric clinical phenotype, frequently characterised by upper respiratory tract involvement [2]. Both GPA and microscopic polyangiitis (MPA), the second most prevalent subtype, can have lower respiratory and renal involvement alongside frequently reported constitutional symptoms such as weight loss and fever [2]. c-AAV is often organ threatening, and the therapeutic regimens traditionally used to induce and maintain remission are extrapolated from adult data and carry significant toxicity. The foundation of induction treatment has been high-dose and prolonged glucocorticoid therapy. However, these have important short-term impact on physical and psychological health, along with less certain but potentially important long-term adverse effects on bone and cardiovascular health. Avacopan is a new treatment that has been demonstrated to decrease the cumulative glucocorticoid dosing for induction among adults with AAV [3] and potentially also as an adjunct treatment for refractory disease [4]. The mechanism of action is as a complement 5a receptor-1 antagonist, stopping the C5 molecule from binding to the receptor and potentiating neutrophil chemoattraction and activation, a key pathogenic step in development of AAV [5].

The updated European Alliance of Associations for Rheumatology AAV guidelines [6] recommend considering the addition of avacopan for induction of remission in adults. In Australia, avacopan was approved by the Therapeutic Goods Administration in 2023 and subsequently made available via government subsidy in 2024 without restriction by age; thus, access to this medication for children has been greater than in other countries. We report on the safety and efficacy of avacopan in 8 adolescents with AAV.

## CASE SERIES

The Table outlines the demographics, disease features, treatment, and outcomes of the 8 patients, identified by survey of Australian paediatric rheumatology and nephrology centres. The median age was 15.5 years (range, 14-18 years). All patients had a positive ANCA; 87% were PR3 positive. Six patients (75%) had renal involvement at presentation, 2 with acute renal failure. Three patients (37.5%) had haemoptysis, suggestive of pulmonary capillaritis. All patients received rituximab and/or cyclophosphamide (4 patients receiving both) at induction, 1 of whom also required plasmapheresis for pulmonary haemorrhage with respiratory failure. All patients received pulsed intravenous methylprednisolone (3 days of 10-30 mg/kg; maximum, 1 g), followed by high-dose oral prednisolone (1 mg/kg/d or maximum 60 mg except for patient 1 who was escalated to 2 mg/kg/d for nephrotic syndrome), with weaning regimens determined by treating physician. Avacopan was started at a median of 2 months (range, 0-19 months) for severe or incomplete disease control and/or to facilitate steroid weaning, with 1 patient starting at induction. Five of the patients had good response to addition of avacopan (2 with predominately sinus/eye disease, 1 with severe pulmonary disease, and 2 with predominately kidney disease, 1 of whom also had significant joint involvement). Having achieved complete disease control (determined by physician assessment and normalisation of laboratory markers), 3 patients were able to wean off oral glucocorticoids following avacopan introduction (2 at 4 months; 1 at 5 months), and 2 were able to wean to low-dose oral steroids. Three patients’ disease (2 with predominately renal disease and the other with pansinusitis) demonstrated progression despite use of avacopan with end-organ damage (end-stage renal failure requiring dialysis and pachymeningitis).

**Table:** Baseline characteristics, treatment course, and clinical outcomes of paediatric patients with ANCA-associated vasculitis treated using avacopan

Demographics/clinical type	ANCA (U/mL) at presentation (normal values: PR3 < 2, MPO < 3.5)	eGFR^a^ (mL/min/1.73 m^2^)	Presenting features	Adjunct treatment	Timing and rationale for avacopan/dose/steroid daily dose at time of avacopan introduction	Disease trajectory
Patient 1: 14-y-old man; 47 kg; Caucasian; GPA	PR3, 259	60 (at diagnosis) 30 (starting avacopan)	Acute renal failure and nephrotic range proteinuria; arthritis; rash; anaemia; lung granuloma; epistaxis	Induction IVMP 1 g × 3 d RTX 1 g fortnightly × 2 Oral prednisolone Maintenance AZA and MMF	Started at 1 mo (fail to wean steroids and renal flare [nephrotic syndrome]) 30 mg twice daily per oral 80 mg oral prednisolone	Initial improvement with ability to wean steroids Ongoing steroid dependence (prednisolone ≥1 mg/kg/d to maintain stable kidney function) and worsening renal failure; PD at 11 mo postdiagnosis Extra renal disease quiescent Ceased avacopan at 10 mo (inefficacy)
Patient 2: 18-y-old female; 62 kg; Caucasian; GPA	PR3, 62	>90	Acute pulmonary haemorrhage with T1RF; arthralgia; necrotising gingivitis; rash; weight loss	Induction IVMP 1 g × 3 d PLEX × 7 courses RTX 1 g fortnightly × 2 Maintenance Oral prednisolone	Started at 3 mo (incomplete disease control) 30 mg twice daily per oral 7.5 mg oral prednisolone	Disease remission on treatment Weaned off steroids as of 4 mo postdiagnosis Continues on avacopan at 11 mo
Patient 3: 16-y-old female; 56 kg; Caucasian; GPA	PR3, 140	53 (at diagnosis) 46 (starting avacopan)	Haematuria/proteinuria; cough; airspace opacity; arthralgia; haemoptysis; epistaxis; rash; abdominal pain; diarrhoea	Induction IVMP 1 g × 3 d RTX 1 g fortnightly × 2 CYC 500 mg × 2 Maintenance Oral prednisolone	Started at 2 mo (persistently elevated SeCr) 30 mg twice daily per oral 50 mg oral prednisolone	Normalisation of SeCr (eGFR > 90) Prednisolone tapered to 5 mg by 6 mo postdiagnosis—continued to 12 mo then ceased Avacopan given for 12 mo then ceased Continues on RTX
Patient 4: 17-y-old male; 53 kg; Caucasian; GPA	PR3, 200	>90 (at diagnosis) 29 (after first RTX) 57 (starting avacopan)	Arthralgia/arthritis (severe—needing opiates); haematuria/proteinuria; fever; myalgia; weight loss; oral ulcers/nasal crusting; cough; haemoptysis; cavitating lung lesions on CT chest	Induction/maintenance IVMP 1 g × 3 d Oral prednisolone RTX 1 g fortnightly × 2 CYC 800 mg monthly × 2	Started 6 wk (persistently elevated SeCr) 30 mg twice daily per oral 50 mg oral prednisolone	Normalisation of SeCr (eGFR > 90) Prednisolone tapered to 5 mg daily by 6 mo post diagnosis—continues Continues on RTX and avacopan at 6 mo
Patient 5: 14-y-old female; 64.5 kg; Caucasian; GPA	PR3, >1000	>90	Severe pansinusitis (earlobe abscess/postauricular sinus); arthritis; rash; fevers; bilateral cavitating lung lesions (no haemoptysis); anaemia; glomerulonephritis; hypertension	Induction IVMP 1 g × 3 d RTX 1 g fortnightly × 2 Oral prednisolone CYC 1 g monthly × 6 Maintenance IVMP 1 g × 3 d Oral prednisolone AZA	Started at 19 mo postdiagnosis (offered at 3 mo for incomplete control/steroid toxicity/high PR3) 30 mg twice daily per oral 5 mg oral prednisolone	Weaned off steroids 6½ mo after avacopan commenced Continues on RTX 6 mo and avacopan at 2 y (5 mo avacopan treatment)
Patient 6: 17-y-o male; 104 kg; Caucasian; GPA	PR3, 136	>90	Upper respiratory tract symptoms (nasal congestion, aural fullness); sensorineural hearing loss; dyspnoea; haemoptysis; low-grade haematuria/proteinuria; fever	Induction/maintenance IVMP 1 g × 3 d (received on 3 occasions over first 12 mo) Oral prednisolone RTX 1 g fortnightly × 2 IVIG × 1 CYC 1 g monthly × 6	Started at 6 mo (failure to wean steroids, progressive nasopharyngeal involvement/symptoms and development of pachymeningitis) 30 mg twice daily per oral 20 mg oral prednisolone	Marginal initial improvement, failure to wean steroids with persistent nasopharyngeal disease, pachymeningitis and rising PR3 titres. Ceased avacopan at 13 mo (inefficacy)
Patient 7: 15-y-old female; 62.5 kg; Hispanic; GPA	PR3, 42	>90	Headaches/sinusitis; right eye proptosis; epistaxis; cough; multifocal cavitating pulmonary nodules	Induction IVMP 1 g × 3 d RTX 375 mg/m^2^ weekly × 4 Maintenance Oral prednisolone	Started at 2 mo to facilitate more rapid steroid wean 30 mg twice daily per oral 50 mg oral prednisolone	Complete clinical/biochemical remission on monotherapy Weaned off steroids at 5 mo (delayed due to medication compliance) Ceased avacopan (self-ceased at 6 mo)
Patient 8: 14-y-old male; 53 kg; Turkish; MPA	MPO, 46	24	Severe anaemia; acute renal failure; diffuse cresenteric glomerulonephritis; subnephrotic proteinuria; microhaematuria	Induction/maintenance IVMP 500 mg × 3 d Oral prednisolone CYC 500 mg fortnightly × 6 PLEX × 5 courses RTX 500 mg (further 6-wk course oral prednisolone)	Started in first week due to severe anaemia and acute renal failure 30 mg twice daily per oral 50 mg oral prednisolone	Initially weaned steroids over 1 mo Deteriorating renal function + new onset haemoptysis at 2 mo; progression to HD and then PD Continues on avacopan at 6 mo

ANCA, antineutrophil cytoplasmic antibody; AZA, azathioprine; CYC, cyclophosphamide; eGFR, estimated glomerular filtration rate; GPA, granulomatosis with polyangiitis; HD, haemodialysis; IVMP, intravenous methylprednisolone; MMF, mycophenolate mofetil; MPA, microscopic polyangiitis; MPO, myeloperoxidase; PD, peritoneal dialysis; PLEX, plasma exchange; PR3, proteinase-3 antibody; T1RF, type 1 respiratory failure; RTX, rituximab; SeCr, serum creatinine.

a eGFR calculated using full-age spectrum equation.

The patients were on avacopan for a median of 6 months (range, 5-13 months). No patient had significant infection after commencement, noting 2 patients having previously had significant infections while on alternate immunosuppression including moderate dose oral steroids (one influenza A and cellulitis/osteomyelitis and another with herpes simplex virus 1 reactivation). One patient reported significant fatigue after starting avacopan. No other adverse effects were reported or observed.

## DISCUSSION

To the best of our knowledge, this case series outlines the largest cohort of adolescents with AAV to receive avacopan. Five of 8 patients demonstrated a positive response to avacopan initiation in disease trajectory and/or subsequent ability to tolerate weaning of glucocorticoid therapy.

The observed treatment effect occurred despite the cohort being skewed towards severe disease. With exception of patient 8 who was commenced on avacopan at diagnosis, all patients had demonstrated a suboptimal response to conventional treatment regimens, and in many, avacopan was being used as an adjunct agent to high-dose glucocorticoids and other immunosuppressant agents until an improving disease trajectory was observed. This approach is similar to real-world data for avacopan use for adult patients with AAV [4] but contrasts with randomised trials thus far [3] in that enrolled participants had comparatively less severe disease and who were receiving avacopan in combination with a rapid glucocorticoid wean. Patient 8 did wean off glucocorticoids within a month but restarted at 2 months in the context of deteriorating renal function and new-onset haemoptysis. Although several adult studies have demonstrated efficacy and safety of avacopan, paediatric data are scarce. Two separately reported c-AAV cases showed utility of addition of avacopan to intense immunosuppressive regimens. A 12-year-old girl with severe disease at diagnosis including need for haemodialysis had rapid renal improvement and tolerated complete wean of oral prednisolone within 1 month of avacopan initiation [7]. A 19-year-old woman with severe refractory pulmonary disease and steroid dependence, diagnosed at 9 years old, not only had marked improvement in clinical state but also was able to wean off steroids almost 3 years after commencing avacopan [8].

Late kidney benefit was seen in another paediatric case report of an 11 year old with C3 glomerulonephritis, with marked improvement of previously refractory proteinuria with open-label avacopan use after 26 weeks of placebo in trial. Repeated effect was again seen after a spike in proteinuria following mandatory cessation at completion of the trial, which improved on recommencement of avacopan via compassionate access [9].

No significant risk signals were observed among our patients despite use of the adult dosing (30 mg twice daily) among all. One patient reported fatigue after avacopan initiation, which did not require cessation or further intervention. No opportunistic or severe infections were observed during treatment. Importantly, no patient had drug-induced liver injury observed from avacopan, noting 1 patient delayed commencement to allow for normalisation of liver function prior. Adult studies and a single paediatric case report have highlighted a small but significant risk of drug-induced liver injury with avacopan, especially in the Japanese population [10]. In the paediatric case, a 10-year-old boy developed significant hepatotoxicity on day 31 after avacopan introduction, which subsequently resolved with cessation of the drug [11]. In our cohort, 1 patient self-ceased avacopan after 6 months for unclear reasons; however, he was clinically in remission off glucocorticoids at this time although did have rising PR3 titres (having previously been negative on treatment).

The authors acknowledge the small numbers, the variability of presentations and treatment regimens, and retrospective nature of data collection of this series, which limits the generalisability of this cohort more broadly. The ultrarare nature of c-AAV is highlighted by the only open trial of avacopan in this group (NCT06321601), aiming to enrol 20 children over an 8-year period. These data are not expected prior to 2030. Hence, as with other orphan diseases (especially paediatric), real-world reports are important; the goal being to detect effect modification or difference in risk compared with larger clinical trial populations.

## CONCLUSION

In this cohort of children with severe and/or refractory AAV, the use of avacopan was associated with improvement in disease manifestations for most. No opportunistic or serious infections were observed following avacopan introduction, nor liver function test derangement. Further research in childhood AAV to better delineate patient groups most likely to benefit and to demonstrate long-term safety will further aid to guide physician decision making.

## Funding

None.

## Patient consent for publication

Patient consent gained prior to inclusion in this series.

## Ethics approval

**Provenance and peer review**

## CRediT authorship contribution statement

**Anne Sage:** Writing – review & editing, Writing – original draft, Data curation, Conceptualization. **Jonathan Akikusa:** Writing – review & editing, Data curation. **Sai Min Htay:** Writing – review & editing, Data curation. **Cassandra Cardenas-Brown:** Writing – review & editing, Data curation. **Gowri Manoharan:** Writing – review & editing, Data curation. **Anne Durkan:** Writing – review & editing, Data curation. **Nicholas Larkins:** Writing – review & editing, Writing – original draft, Data curation, Conceptualization.

## Competing interests

None.
